# Comparison of percutaneous vertebroplasty and percutaneous vertebroplasty combined with pediculoplasty for Kümmell’s disease: a retrospective observational study

**DOI:** 10.1186/s13018-023-03957-5

**Published:** 2023-06-29

**Authors:** Teng Liu, GuoNing Gu, ChenGuang Zhan, ShunCong Zhang, YongChao Tang

**Affiliations:** 1 The 1st institute of Clinical Medicine, Guangzhou University of Chinese Medicine, Guangzhou, Guangdong China; 2 Spine Surgery Department, The First Affiliated Hospital of Guangzhou University of Chinese Medicine, Guangzhou, China

**Keywords:** Kümmell’s disease, Vertebroplasty, Pediculoplasty, Bone cement loosening

## Abstract

**Background:**

To investigate the clinical outcomes of percutaneous vertebroplasty (PVP) versus percutaneous vertebroplasty combined with pediculoplasty (PVP-PP) for Kümmell’s disease (KD).

**Methods:**

Between February 2017 and November 2020, 76 patients with KD undergoing PVP or PVP-PP were included in this retrospective study. Based on the PVP whether combined with pediculoplasty, those patients were divided into PVP group (*n* = 39) and PVP-PP group (*n* = 37). The operation duration, estimated blood loss, cement volume, and hospitalization stays were recorded and analyzed. Meanwhile, the radiological variations including the Cobb’s angle, anterior height of index vertebra, and middle height of index vertebra from X-ray were recorded preoperatively, at 1 days postoperatively and the final follow-up. The visual analogue scale (VAS) and Oswestry disability index (ODI) were also evaluated. Preoperative and postoperative recovery values of these data were compared.

**Results:**

The two groups showed no significant difference in demographic features (*p* > 0.05). The operation time, intraoperative blood loss, and time of hospital stay revealed no sharp statistical distinctions either (*p* > 0.05), except that PVP-PP used more bone cement than PVP (5.8 ± 1.5 mL vs. 5.0 ± 1.2 mL, *p* < 0.05). The anterior and middle height of vertebra, Cobb’s angle, VAS, and ODI was observed a little without significant difference between the two groups before and 1 days postoperatively (*p* > 0.05). Nevertheless, ODI and VAS scores decreased significantly in the PVP-PP group than in the PVP group at follow-up (*p* < 0.001). The PVP-PP group exhibited a slight amelioration in Ha, Hm, and Cobb's angle when compared to the PVP group, displaying statistical significance (*p* < 0.05). No significant disparity in cement leakage was observed between the PVP-PP and PVP groups (29.4% vs. 15.4%, *p* > 0.05). It is worth noting that the prevalence of bone cement loosening displayed a remarkable decrement within the PVP-PP group, with only one case recorded, as opposed to the PVP group's seven cases (2.7% vs. 17.9%, *p* < 0.05).

**Conclusions:**

Both PVP-PP and PVP can relieve pain effectively in patients with KD. Moreover, PVP-PP can achieve more satisfactory results than PVP. Thus, compared with PVP, PVP-PP is more suitable for KD without neurological deficit, from a long-term clinical effect perspective.

## Background

Kümmell’s disease (KD) is a distinct form of osteoporotic vertebral compression fracture that predominantly affects the elderly population [1]. In 1895, Dr. Hermann Kümmell, a German surgeon, described a clinical syndrome characterized by the gradual manifestation of progressive painful kyphosis following an asymptomatic phase of months or years after minor spinal trauma, leading to a gradual collapse of the vertebra, dynamic instability, progression to kyphosis with prolonged back pain, and potential paraparesis [2]. Consequently, the management of KD necessitates the implementation of a proactive therapeutic approach.

**Fig. 1:**
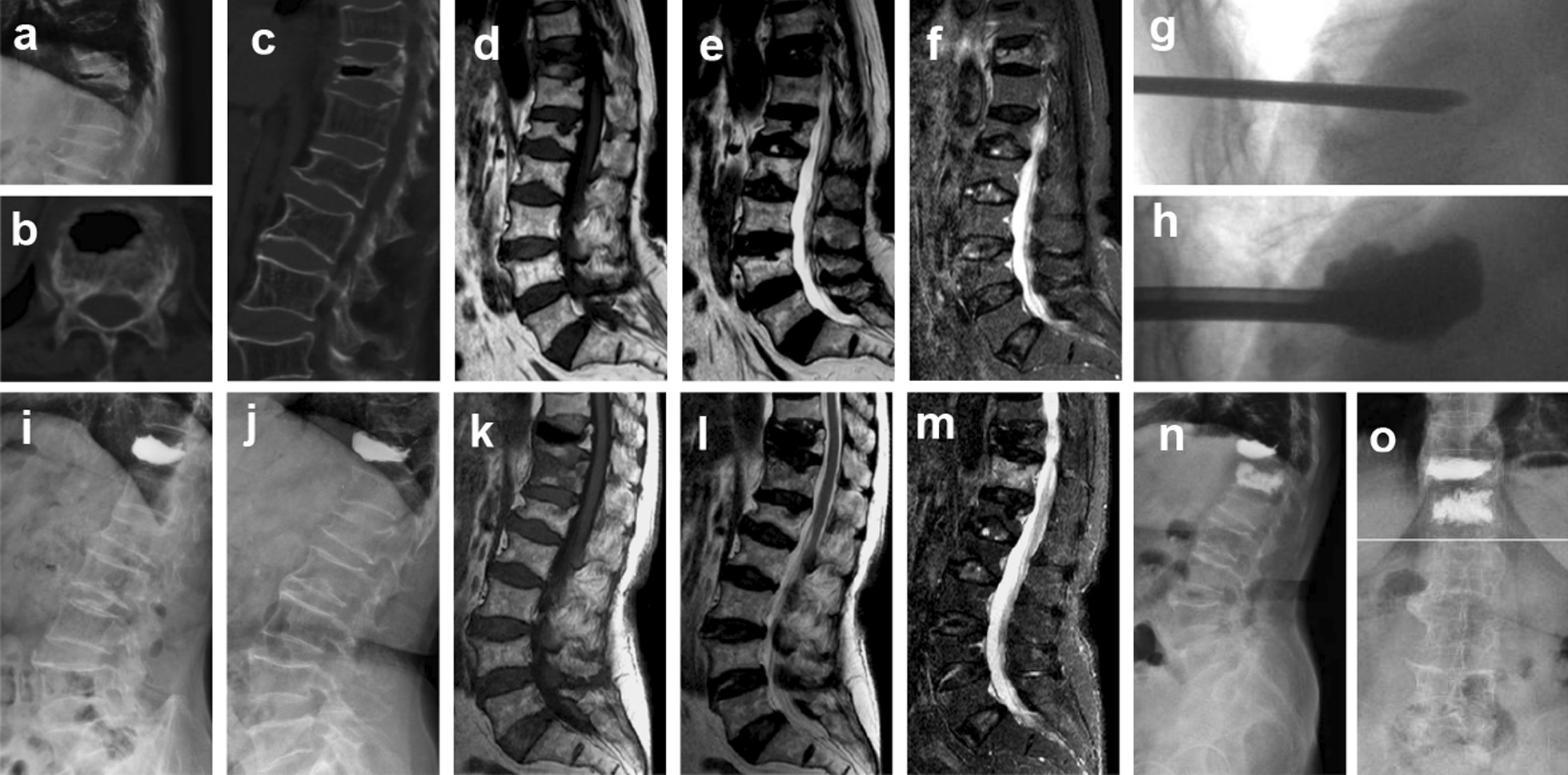
Treatment of Kümmell’s disease via percutaneous vertebroplasty (PVP). A female aged 73 years complained of low back pain and discomfort for 6 months. Preoperative lateral X-ray suggested a reduction in the height of the T11 vertebral body **a** Preoperative computed tomography (CT) revealed dehiscence of the anterior middle region of vertebra T11 and the formation of a vacuum fissure within the vertebra **b, c** Preoperative magnetic resonance imaging confirmed that the T11 vertebral body was a fresh fracture. The vacuum fissure in the vertebral body also evidenced a fluid signal **d–f** The patient underwent PVP. Intraoperative bone cement injection was monitored using X-ray fluoroscopy **g** Intraoperative lateral X-ray after PPP **h** An X-ray taken on the first day after surgery revealed that the bone cement was evenly distributed in the vertebral body **i** An X-ray taken on the 3 years after surgery revealed that the bone cement forward migration and lateral fusion with the adjacent vertebral body, the height of the T12 vertebral body was a reduction **j** Preoperative magnetic resonance imaging confirmed that the T12 vertebral body was a fresh fracture **k–m** A postoperative lateral and anteroposterior X-ray after PVP **n, o**

**Fig. 2:**
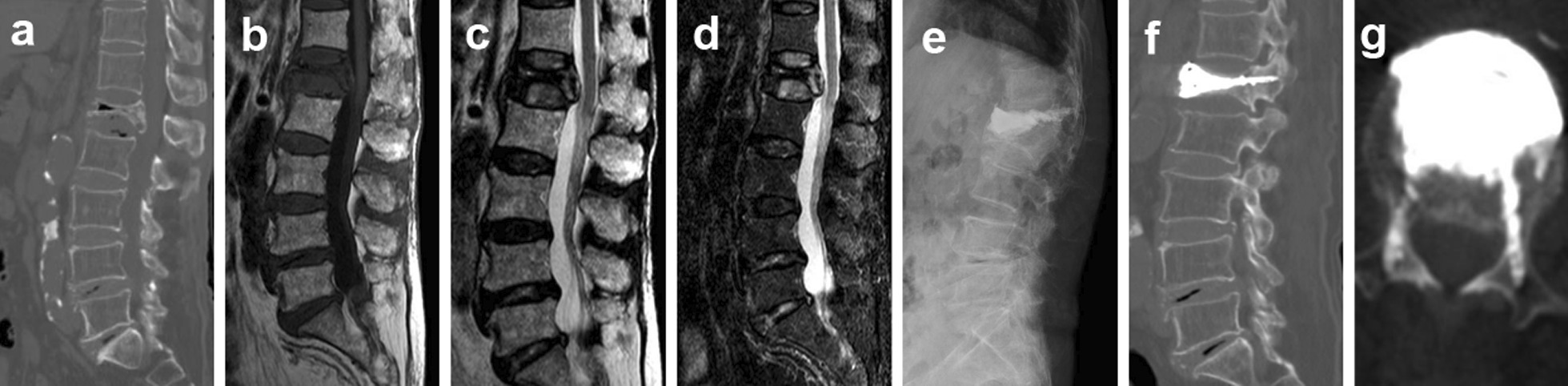
Treatment of Kümmell’s disease via percutaneous vertebroplasty combined with pediculoplasty (PVP-PP). A male aged 80 years complained of low back pain and discomfort for 5 months. Preoperative computed tomography (CT) revealed dehiscence of the anterior middle region of vertebra L1 and the formation of a vacuum fissure within the vertebra (**a**). Preoperative magnetic resonance imaging confirmed that the L1 vertebral body was a fresh fracture. The vacuum fissure in the vertebral body also evidenced a fluid signal (**b-d**). An X-ray taken on the first day after surgery revealed that the bone cement was evenly distributed in the vertebral body and the pedicle (**e**). A postoperative CT scan showed that the bone cement was evenly distributed in the vertebral body and bilateral pedicle without leakage into the vertebral canal (**f, g**)

Percutaneous vertebroplasty (PVP) has been previously proven to be an effective method for the treatment of KD and has the characteristics of minimally invasive, fast pain relief, and fast postoperative recovery [3]. Despite their effectiveness, these methods have been linked to potential issues such as bone cement leakage and the possibility of long-term loosening or displacement of the cement (or even dislodgement) [4]. To prevent cement loosening, previous studies have shown that percutaneous vertebroplasty combined with pediculoplasty (PVP-PP) has good clinical efficacy in the treatment of KD [5]. The present study aimed to compare radiological and clinical outcomes of PVP and PVP-PP in the treatment of KD (Figs. 1 and 2).

## Methods

### Patient characteristics

This retrospective study analyzed 76 patients with neurologically intact osteoporotic KD from February 2017 to November 2020, of which 37 underwent PVP-PP and 39 underwent PVP. The related data of patients are shown in Table 1. Informed consent was obtained from all participants. The retrospective study comprised 23 male and 53 female with a mean age of 77.0 years (range 61–101 years) and a mean disease duration of 5.7 months (range 1–16 months). There were no significantly differences between the PVP and PVP-PP groups in sex, age, and duration of disease (Table 1; *P* > 0.05).

**Table 1:** Demographic and perioperative data of patients

Characteristic	PVP-PP group	PVP group	*P*
Age,years (mean ± SD)	76.0 ± 6.1	77.8 ± 7.6	0.263
Male/female (No.)	12/25	11/28	0.688
Course of disease (months)	5.6 ± 2.5	5.8 ± 2.6	0.772
BMD (T-score)	− 3.3 ± 1.5	− 3.6 ± 0.7	0.415
Follow-up (months)	28.7 ± 6.8	29.3 ± 6.7	0.695
Estimated blood loss (ml)	15.3 ± 4.3	15.1 ± 3.8	0.778
Cement volume (ml)	5.8 ± 1.5	5.0 ± 1.2	< 0.05
Operation duration (min)	37.8 ± 3.7	36.2 ± 4.0	0.077
Hospitalization stays (days)	2.5 ± 0.7	2.5 ± 0.8	0.541

*PVP* Percutaneous vertebroplasty, *PP* Pediculoplasty, *BMD* Bone mineral density

### Inclusion and exclusion criteria

The inclusion criteria were (1) preoperative diagnosis of KD without neurological symptoms and postoperative follow-up for at least 24 months; lumbar or hip bone mineral density (BMD) with T ≤ − 2.5 SD; (2) PVP or PVP-PP was used with X-ray of the thoracic or lumbar spine before, 1d and at the last follow-up; (3) those with lesions in a single vertebral body.

The exclusion criteria were (1) patients with a primary tumors of the spine, spinal metastases, spinal tuberculosis, vertebral compression fractures caused by septic infection of the spine; (2) fresh vertebral compression fractures or burst fractures from high-energy injuries; (3) those with severe vertebral body compression rendering puncture impossible.

### Surgical techniques

#### PVP group

All of the operations were performed under local infiltration anesthesia. The patient was placed in a prone position, followed by routine disinfection, towel laying, and unilateral or bilateral pedicle puncture. The upper chest and ilium were padded with soft pillows, the chest and abdomen are suspended in the air, and postural repositioning was attempted by pressing on the fracture site. The pedicle of the injured vertebral body was located using the C-arm X-ray machine, the outer and upper side of the pedicle of the injured vertebra as the puncture point was selected, and the puncture needle was inserted into the vertebral body; the fluoroscopy needlepoint reached the posterior edge of the vertebral body, and the anteroposterior fluoroscopy needlepoint was located on the medial wall of the pedicle. During the procedure of lateral fluoroscopy, the tip of the puncture needle was halted upon reaching the anterior middle third of the vertebral body, without entering the spinal canal. Anteroposterior fluoroscopy confirmed that the puncture needle tip was located between the medial pedicle and the spinous process. The bone tissue was collected for pathological examination. During the bone cement wire drawing stage, high-viscosity bone cement was injected slowly. After the intravertebral vacuum cleft (IVC) was filled satisfactorily and diffused in the injured vertebra under fluoroscopy, the injection of the bone cement was stopped, and the puncture needle was removed. The photographs of actual operation are shown in Fig. 1.

### PVP-PP group

**Fig. 3:**
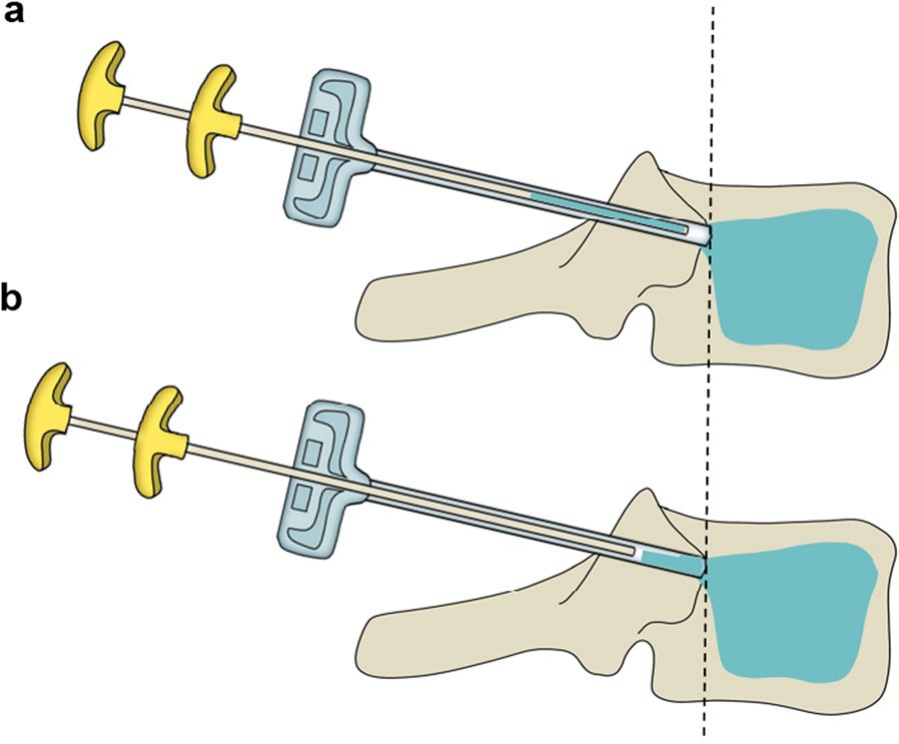
The puncture working cannula was retracted to the posterior 1/4 of the vertebral body (**a**). The bone cement in the working channel and that in the vertebral arch were connected to form a monolithic structure (**b**)

Initial steps were similar to those of PVP. After the diffusion and distribution of the bone cement were satisfied under fluoroscopy, the puncture working cannula was retracted to the posterior 1/4 of the vertebral body, and a small amount (0.1 ml at a time) of the bone cement was injected late in the drawing phase, and while keeping the outer working cannula immobile, the puncture needle to the posterior part of the midpoint of the vertebral arch was withdrawn while pushing the bone cement injection, the working channel was rotated after the bone cement had cured to ensure that the working channel was separated from the bone cement, so that the bone cement in the working channel and that in the vertebral arch were connected to form a monolithic structure. The schematic diagram and photographs of actual operation are shown in Figs. 2 and 3.

The surgical instruments of PVP and PVP-PP were obtained from Dragon Crown Medical (Shandong Province, China), Inc. The patient’s vital signs were monitored throughout the operation. Close attention was paid to the sensation and movement of the patient’s lower limbs during puncture and injection of the bone cement to ensure no spinal cord injury or compression. The patient was allowed to rest in bed for 24 h after the operation. The patient was then allowed to get out of bed after the postoperative X-ray examination was satisfactory, received anti-osteoporosis treatment at the same time and was discharged three days after the operation.

### Outcomes

The operation duration, intraoperative blood loss, time of hospital stay and the volume of bone cement injected in the two groups were recorded. Based on the imaging examination, incidence of cement leaks and cement loosening, Cobb’s angle (defined by the upper endplate of the first vertebra above the diseased one and the lower endplate of the first vertebra below the diseased one), the anterior and middle height of vertebra were measured before and after surgery. The visual analog scale (VAS), ranging from 0 (representing absence of pain) to 10 (indicating the most severe pain), was to assess the intensity of low back pain. Concurrently, the Oswestry Disability Index (ODI), utilizing a scale ranging from 0% (denoting optimal functional state) to 100% (signifying the most compromised functional state), to evaluate the extent of improvement in patients quality of life. Measurements were independently carried out by two researchers, and the mean value was used.

### Statistical analysis

SPSS 25.0 statistical software was used for all analyses. Data were expressed as means ± standard deviations. The before-and-after comparisons of the injured vertebrae Cobb’s angle, injured vertebrae anterior margin and midline height, VAS, and ODI were performed by paired-sample *t*-test. A *p*-value of < 0.05 was considered statistically significant.

## Results

**Fig. 4:**
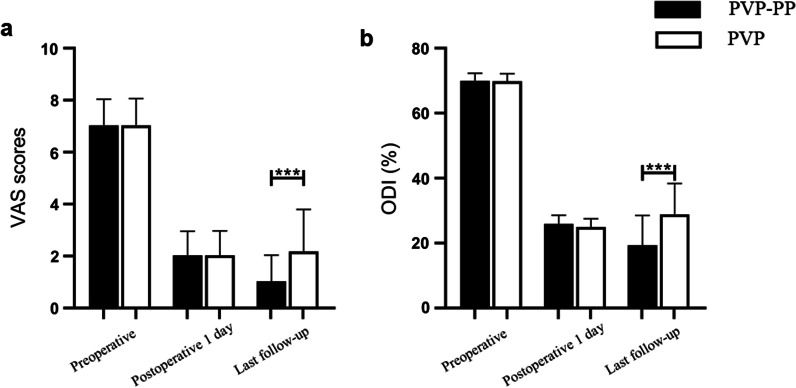
A comparison of VAS scores preoperatively, the one day after the operation and at the final follow-up (**a**). A comparison of ODI preoperatively, the one day after the operation and at the final follow-up (**b**)

The study encompassed a total of 76 eligible patients, comprising 37 patients in the PVP-PP group and 39 patients in the PVP group. Analysis revealed no significant disparities in preoperative demographic characteristics between the two groups (*p* > 0.05), except for a notable distinction in the utilization of bone cement, where the PVP-PP group used more bone cement compared to the PVP group (5.8 ± 1.5 mL vs. 5.0 ± 1.2 mL, *p* < 0.05) (Table 1). No statistically significant difference in the improvement of VAS, ODI, anterior and middle height of index vertebra, and Cobb’s angle was found between the two groups of patients before and 1 days after surgery (*p* > 0.05) (Tables 2 and 3, Fig. 4). However, the VAS, ODI, anterior and middle height of index vertebra were significantly better in the PVP-PP group than in the PVP group (*p* < 0.05). No significant disparity in cement leakage was observed between the PVP-PP and PVP groups (29.4% vs. 15.4%, *p* > 0.05), and the leakage of bone cement did not cause serious complications (Table 4). There were 1 and 7 cases of bone cement loosening in the PVP-PP and PVP groups, respectively (2.7% vs. 17.9%, *p* < 0.05) (Table 4). During follow-up, no other vertebral fractures were observed in either group.

**Table2:** Comparison of VAS scores for back pain and ODI

Parameters	PVP-PP group	PVP group	*P*
*VAS scores*			
Preoperative	7.03 ± 1.01	7.03 ± 1.04	0.995
Postoperative	2.03 ± 0.93	2.03 ± 0.93	0.995
Final follow-up	1.03 ± 1.01	2.18 ± 1.62	< 0.001
*ODI scores*			
Preoperative	69.97 ± 2.30	69.80 ± 2.35	0.754
Postoperative	24.98 ± 2.50	25.87 ± 2.69	0.142
Final follow-up	19.34 ± 9.18	28.85 ± 9.50	< 0.001

*VAS* Visual analogue scale, *ODI* Oswestry Disability Index

**Table 3:** Comparison of radiological evaluation results

Variable	PVP-PP group	PVP group	*P*
*The cobb angle* (°)			
Preoperative	20.67 ± 9.56	20.62 ± 9.60	0.979
Postoperative	13.28 ± 8.82	13.21 ± 8.01	0.971
Final follow-up	13.72 ± 9.00	18.14 ± 9.12	< 0.05
*Anterior height of fractured vertebra* (cm)			
Preoperative	1.36 ± 0.36	1.36 ± 0.39	0.997
Postoperative	1.90 ± 0.35	1.88 ± 0.29	0.803
Final follow-up	1.78 ± 0.35	1.61 ± 0.34	< 0.05
*Middle height of fractured vertebra* (cm)			
Preoperative	1.27 ± 0.33	1.26 ± 0.32	0.901
Postoperative	1.87 ± 0.24	1.84 ± 0.27	0.634
Final follow-up	1.70 ± 0.26	1.56 ± 0.29	< 0.05

**Table 4:** Comparison of complications

Variable	PVP-PP group	PVP group	*P*
Cement leakage (No.)	10	6	0.213
Bone cement loosening (No.)	1	7	< 0.05

## Discussion

KD is defined as avascular vertebral osteonecrosis. It may lead to back pain, cosmetic deformity, and risk of neurological compromise or even mortality [6]. In recent years, surgical intervention has become the preferred method for the treatment of KD, and conservative treatment modalities are usually unsatisfactory due to their poor efficacy and the inability of patients to tolerate persistent, progressive pain [7]. PVP can quickly relieve pain and restore vertebral height and has obvious short-term effect for treating painful KD [8]. However, a subset of patients may encounter the untoward phenomenon of cement loosening accompanied by a subsequent reoccurrence of back pain subsequent to the procedure [9].

Recently, with the widespread use of vertebroplasty, bone cement loosening has rarely been reported as one of the complications following vertebroplasty. According to previously published reports, the incidence of bone cement loosening (or even dislodgement) after vertebroplasty is estimated to be between 0.51 and 25% [9–11]. However, the precise mechanism underlying bone cement loosening remains elusive. Several risk factors for bone cement loosening have been identified, including intravertebral pseudarthrosis, anterior cortical defect, endplate damage, and insufficient amount of bone cement injection [12, 13]. In particular, bone cement loosening is closely associated with intravertebral pseudarthroses, leading to instability. Huang et al. [14] found by pathological examination that KD patients with intravertebral cleft (IVC) form a pseudomembrane, which affects the diffusion of bone cement into the surrounding trabeculae during vertebroplasty, causing the bone cement to be confined only to the IVC, making it difficult for the bone cement to maintain stability in the vertebral body, eventually leading to loosening (or even dislodgement) of the bone cement, the collapse of the vertebral body, and poor therapeutic efficacy. Lane et al. [15]found that an insufficient volume of bone cement during vertebral body reinforcement caused the bone cement to not completely fill the bone cement gap and maintain the stability of the vertebral body. Therefore, PVP should be performed with caution for complicated KD with dynamic instability.

Reducing the incidence of cement displacement in KD, some scholars propose a combination of posterior transpedicular internal fixation and vertebroplasty to reduce the incidence of complications [16]. However, the procedure is invasive and requires general anesthesia, increasing the risk of surgery in elderly patients [17]. Recent studies reported that PVP-PP is derived from PVP based on the same pathologic mechanism; after filling the vertebral body with bone cement, the cement is then injected into the pedicle to anchor the bone cement in the vertebral body, creating a three-column strengthening for individuals diagnosed with stage I and II KD with an intact posterior wall of the vertebral body and intact arch cortices on at least one side [18]. Wang et al. [5] treated 87 KD patients without neurological symptoms, and two-year postoperative follow-up data showed that PVP-PP had good short-term and medium-term therapeutic effects, effectively prevented bone cement loosening and achieved rapid pain relief, satisfactory vertebral body height restoration, and kyphotic deformity improvement. Liu et al. [18] applied PVP-PP in the treatment of 39 patients with KD and described the technique in detail, with minimal trauma, rapid recovery, and firm cement fixation. However, PVP-PP is operationally demanding in terms of the pedicle anchoring technique and has a relatively increased risk of cement leakage.

PVP-PP is the injection of bone cement into the pedicle based on PVP, and its operation is relatively difficult [19]. Based on the findings of the present study, the main points of the PVP-PP technique operation can be summarized as follows. ① The patient may be given appropriate analgesic medication first to reduce pain, the integrity of the posterior longitudinal ligament is assessed preoperatively when the posterior longitudinal ligament is completely ruptured, relying on the tension of the posterior longitudinal ligament to reposition the fracture fragment protruding into the spinal canal is no longer possible, and postural repositioning is often considered contraindicated at this time. ② During the process of pediculoplasty, the selected bone cement should be in the late drawing stage or early dough stage. ③ Proceed slowly and uniformly under strict fluoroscopy, inject a small amount (0.1 ml each time) of bone cement in the late drawing stage, push the injection while withdrawing the puncture needle, and several times with lateral fluoroscopy to ensure the consistent and homogeneous distribution of the bone cement within the entire pedicle. Once the bone cement is found to move perpendicular to the direction of the needle, the operation should be suspended. ④ After the cement has cured, the working channel is rotated to ensure that it is separated from the cement, so that the cement in the working channel and that within the pedicle are joined to form a monolithic structure.

The use of PVP-PP in KD patients is controversial in terms of unilateral or bilateral puncture [20]. A unilateral puncture can achieve the same surgical results as a bilateral puncture, with shorter operative time and less trauma, and it is also safe and economical. However, it requires a greater abduction angle to allow the puncture needle to cross or approach the vertebral body midline, increasing the risk of damaging the medial cortex of the arch. Bilateral puncture requires a slightly lower puncture angle and is relatively easy to perform; however, it increases the operative time and the risk of bone cement leakage. In choosing a unilateral or bilateral puncture, the location of the IVC and the integrity of the pedicle can be judged based on preoperative imaging: ① if the IVC is located in the one side or center of the vertebral body and the bilateral pedicle is intact, then the pedicle can be punctured on either side and the bone cement can be pushed; ② if one side of the vertebral body is found to be severely collapsed and the pedicle fracture and the image is blurred, then the puncture can be done on the opposite side of the pedicle; ③ if the IVC is located in both sides of the vertebral body and is not connected to each other, then the puncture can be done via the bilateral.

As the most common complication of vertebroplasty, cement leakage into the intervertebral space produces a pillar effect, which accelerates adjacent disk degeneration, reduces disk cushioning, generates abnormal stresses, and increases the risk of fracture of adjacent vertebrae, and can lead to catastrophic consequences such as nerve compression and pulmonary embolism [21]. The current study showed that PVP-PP had a slightly higher rate of bone cement leakage compared with the PVP technique (29.4% vs. 15.4%, *p* > 0.05), but there was no statistical difference, which could be because PVP-PP compared with PVP increases the risk of bone cement leakage at the pedicle with the addition of the pedicle anchoring technique step. To reduce the risk of bone cement leakage, the following should be considered: ① PVP-PP should be carefully evaluated before the surgery and PVP-PP is not recommended for patients with an incomplete posterior wall of the vertebral body and incomplete medial cortex of the pedicle; ② place the needle tip in the IVC and confine the bone cement to diffuse in the IVC; ③ in patients with anterior cortical defect, a fractionated push-injection technique can be used, in which high-viscosity cement is injected anteriorly to fill the fissure and then a relatively dilute bone cement is injected; ④ pediculoplasty should be performed under local anesthesia to facilitate observation of the patient for neurogenic pain, suggesting potential cement leakage [22].

Some studies have indicated that the amount of bone cement injected is positively correlated with the incidence of bone cement leakage [23]. In patients with KD, the amount of bone cement injected should be as high as possible and adequately fill the cavity to ensure spinal stability and good pain relief, and the ideal vertebroplasty should cover the cleft area and the cancellous bone area on the cephalocaudal side of the cleft. Our study found that insufficient bone cement led to intravertebral instability or vertebral collapse, resulting in poor outcomes, and injection of an adequate amount of bone cement (5.8 ± 1.5 ml) into the injured vertebral fissure resulted in better clinical outcomes at long-term follow-up.

At present, studies exploring the efficacy of PVP-PP and PVP in the treatment of KD patients without neurological symptoms are scarce. Our study showed that both surgical methods had comparable effects on the improvement of the VAS and ODI scores postoperatively, and long-term follow-up PVP-PP was better than PVP. Bone cement loosening was significantly lower in the PVP-PP group than in the PVP group [2.7% (1/37) vs. 17.9% (7/39), *P* < 0.05]. PVP-PP significantly reduced the incidence of long-term complications after PVP because the bone cement in the vertebral body was extended backward through the working channel into the pedicle, which played a specific role in anchoring the front bone cement [18]. Insufficient bone cement can lead to intravertebral instability or vertebrae body collapse, leading to poor curative effect [24]. In our study, IVC was filled with a sufficient amount of bone cement.

Nonetheless, this investigation has several limitations. First, this study is a retrospective analysis, and no method was adopted to ensure unbiased randomization of the two groups. Second, the tailing part of the bone cement is small in volume, and the root connected with the bone cement is relatively weak, and therefore the overall mechanical strength is not as good as vertebroplasty combined with screw fixation in theory. Finally, the number of cases and centers was quite limited. Therefore, more high-quality large-scale randomized controlled trials are needed to validate these preliminary findings.

## Conclusion

In summary, both PVP and PVP-PP are effective in providing pain relief in patients with neurologically intact osteoporotic KD, and they have the advantages of minor trauma, short operation time, and quick recovery. However, compared with PVP, PVP-PP seemed to be more suitable for KD patients without neurological deficits from a long-term clinical effect viewpoint.
